# Reproductive Health Care for Women with Spina Bifida

**DOI:** 10.1100/tsw.2007.304

**Published:** 2007-11-26

**Authors:** Amie B. Jackson, Pamela K. Mott

**Affiliations:** Departments of Physical Medicine and Rehabilitation, University of Alabama at Birmingham School of Medicine, Birmingham, AL, USA

**Keywords:** females, women, disabilities, spina bifida, reproductive health, meningomyelocele, hydrocephalus

## Abstract

Women with spina bifida have unique health care concerns and as the life expectancy of this population increases, they are transitioning from adolescence to womanhood and entering their reproductive years with little information about what to expect. Likewise, their health care providers do not have the benefit of evidence-based research that comprehensively addresses the issues these women may face related to reproduction or aging. Few studies have focused on the effects that spina bifida may have on these women's reproductive systems, nor has attention been paid to the effects that possible reproductive endocrine changes may have on their disability. Needless to say, concerns about sexuality, sexual function, and pregnancy are just as important to these women as they are to their able-bodied counterparts.

### GYNECOLOGICAL ISSUES

#### Menarche

A number of studies[1,2,3,4,5,6,7,8] have demonstrated that sexual maturation often occurs earlier in girls with spina bifida. The percentage of their able-bodied counterparts who undergo menarche at an early age (<12 years of age) is 0.6%[7], while the prevalence of early puberty for girls with spina bifida is 12%[2] to 16%[7]. This difference may be related to the presence of a history of hydrocephalus for girls with spinal bifida, as most studies relate this specific population with precocious puberty; however, studies examining early puberty in the absence of hydrocephalus are lacking. Additionally, the average age of menarche for these girls is often earlier, ranging from 10.9[4] to 11.4[8] years of age as compared to able-bodied girls 12.7 years of age[8]. No specific information is available regarding the qualities of menstruation in women with spina bifida.

### Anatomical Anomalies

Few studies examine specific gynecologic problems or reproductive endocrine dysfunction unique to women with spina bifida. It is known that a few of these women have congenital anomalies of the female reproductive tract, such as bicornate uterus[9]; however, no detailed studies have actually looked at the extent of these and other possible anatomical changes. Depending on the preservation of pelvis floor musculature strength and tone, it is not uncommon for these women to develop a prolapsed uterus at a much younger age. Treatment may be difficult as standard methods may not be efficacious. For example, surgical correction may not be possible due to the pelvic anatomy, and a pessary (or other device) may create pressure points that cause intravaginal mucosal breakdown. Also, if the device is fabricated with latex, it may produce a vaginal allergic reaction in those women sensitive to latex.

Information is so limited about the prevalence of gynecologic conditions in these women that determining whether they have similar and/or dissimilar gynecologic concerns must rely on extrapolations from studies of women with similar disabilities or studies of a merged subgroup of the "physically impaired". Surveys of women with spinal cord injury (SCI)[10,11,12] and multiple sclerosis[13] have provided descriptive data about gynecologic concerns for neurologically and mobility-impaired women. Most are case studies indicating that women with SCI are similar to able-bodied women in terms of menstruation and the ability to conceive. The major menstrual problem women with SCI face is hygienic management due to their immobility. Monthly endocrine fluctuations have been shown to exacerbate spasticity, autonomic fluctuations, and bladder hyper-reflexia following SCI. Whether or not these occur in women with spina bifida is unknown.

### Secondary Conditions

Orthopedic conditions, such as kyphoscoliosis, hip dislocation/subluxation, lower extremity contractures, and pelvic obliquity, are secondary conditions seen in individuals with spina bifida[14,15]. These conditions may physically alter the woman's reproductive anatomy, which may make a gynecologic exam difficult. In addition, positioning on an exam table may require special attention. These issues impact the pelvic examination and assessment, and knowledge of them is vital when these women present with gynecologic concerns. If a satisfactory exam cannot be performed and the woman presents with specific abnormal symptoms related to her reproductive tract, a pelvic CT or ultrasound should be obtained.

Surgical interventions, such as intraperitoneal gynecologic or urologic procedures, may require a unique approach. Consideration of the presence of cerebral spinal fluid (CSF) shunting is important due to a reported 41.4% incidence of shunt complications following these procedures[16]. Although this high incidence was thought to have some association with bowel contamination, these findings may have implications for women with CSF shunts (ventriculoperitoneal or ventriculoatrial) who have intraperitoneal gynecological procedures. Of three women followed in this author's (Jackson) newly established Transitioned Women's Clinic, this complication did not occur. Additional studies are needed to examine the ramifications of pregnancy on shunts.

Another secondary condition of spina bifida has been noted that may have reproductive health ramifications. It is well known that individuals with spina bifida have an increased risk of developing latex allergies[17,18]. Of women with spina bifida, 23% have a natural rubber latex allergy that is postulated to correspond to the number of surgical interventions they have undergone. The belief that an inherent state of atopy was the primary etiology has been declared to be only a minor contributing factor[19]. It is imperative that questions about latex allergies are included in the medical history so that gynecologic examinations, surgical interventions, and use of reproductive health products, such as condoms and diaphragms, are latex free.

### Sexual Response

Little information exists on the effects of spina bifida on the female sexual response. Again, only extrapolation from the SCI data can offer possible neurological correlations for lubrication and orgasm based on spinal cord or peripheral nerve deficits[20,21,22,23,24,25,26]. There are no data on the incidence of sexual dysfunction for women with spina bifida (as defined by DSM IV or the International Consensus Development Conference Standards for the Classification of Female Sexual Dysfunction[27]). It is imperative that full sexual histories be cautiously elicited when treating women with spina bifida due to their possible naiveté and lack of sexual education[28], or in the event of a past history of sexual abuse (which has been reported to be greater in women with disabilities)[29].

Little has been written on the difficulties these women have with sexual function. Difficulties with positioning due to the skeletal and reproductive tract changes challenge the couple to look for new ways to have sexual intercourse. In addition, more planning is required for those women who have neurogenic bowel and bladder, which often necessitates working evacuation of bowel and bladder just before intercourse. Other physical considerations include observance of osteoporosis and possibilities of fractures, pain with intercourse, presence of decubiti, and any respiratory compromise as a result of positioning.

### Birth Control

The need for birth control and what methods are safest for women with spina bifida have not been examined. In an early prevalence study[30], women with physical disabilities who were sexually active reported much lower birth control practices. Many claimed inadequate sexual health education and a lack of understanding of the options available. In one controlled study of women with SCI, a questionnaire methodology looked at their birth control habits. Of 315 women with disabilities and 301 control subjects, the disabled women were less likely to use hormonal contraception (11.7/19.7%), were significantly more likely to have had a hysterectomy (8.9/4.3%), and were significantly more likely to use “no method” of birth control (41.9/33.4%)[31]. Again, these data can only be extrapolated to women with spinal bifida.

While no studies have examined risks and benefits for specific birth control methods for women with spina bifida, the woman and her health care provider should consider the known relative concerns for several forms of birth control. Due to the fact that women with spina bifida often have advanced osteoporosis from disuse and immobility, oral contraceptives composed mostly or completely of progesterone may potentially exacerbate this problem. Similarly, oral contraceptives composed mostly of estrogens have the tendency to promote a hypercoaguable state in women and, due to immobility, this may become more pronounced. Although no evidence exists for any woman with a stable paralysis to be at a greater risk for deep venous thrombosis or pulmonary thromboembolism, caution should be used with this form of birth control. Of course, these forms of birth control should not be used if the woman has the same risk factors as able-bodied women (i.e., active bone disease, smoking, history of headaches, or cardiovascular disorders). Finally, use of intrauterine devices (IUDs) should be avoided if the woman has a history of chronic urinary tract infections, impaired pelvic sensation, or a prolapsed uterus. This precaution lessens the likelihood for this foreign body to cause pelvic infections that may not be detected in a timely manner.

## OBSTETRICAL ISSUES

When contemplating pregnancy, women with spina bifida should consider the changes that will occur. Initial published reports of the obstetrical experiences of these women were single case studies. However, as the population and average age of women with spina bifida have increased[32], more have become pregnant. Although large series of surveys and case studies have contributed to the medical management of these women, standards for their obstetrical care still do not exist. These studies have mostly described pregnancy complications unique to these women as well as medical challenges encountered due to secondary conditions, such as kyphoscoliosis, ventricular shunts, and neurogenic bowel and bladder procedures.

It is well known that all women should have sufficient folate supplementation to lessen the risk having a newborn with spina bifida. Past literature has recommended an intake of 0.4 mg of folate per day for pregnant able-bodied women[33]. More recent recommendations[34] have utilized new standards in measuring the amount and forms of folate required for able-bodied women who become pregnant. These guidelines look at all forms of biologically available folate and recommend 1362 nmol (600 µg) of DFE/d (= dietary folate equivalent per day). This is based on the maintenance of normal red cell folate concentrations required in pregnancy. There are some data, however, to suggest that women with spina bifida should have even larger doses of folate than their able-bodied counterparts. Recommendations[35] are that these women should have 4.0 mg of folate every day before and during pregnancy. Obviously, this question requires urgent investigation.

Retrospective studies[36,37,38,39,40,41] have examined pregnancy experiences of women with spina bifida. Many had neurogenic bowel and bladder, paraparesis, urinary diversions, and comorbidities, such as kyphoscoliosis and previous shunting for hydrocephalus. Common problems associated with urinary tract infections were reported, including symptomatic bacteremia and pyelonephritis. Almost 10% of pregnancies exhibited urological compromise from the growing fetus causing hydronephrosis, intestinal obstruction[37], and renal decline[42]. Intrapartum genitourinary changes contributed to urinary diversion complications and preterm delivery. C-section was common, but, overall, healthy babies were delivered. In one study, however, a woman had three pregnancies that resulted in spontaneous abortion, preterm delivery of a nonviable fetus, and preterm delivery of a healthy infant, respectively[43]. Only one case of maternal death was documented, but appeared unrelated to her disability[44].

These studies also reported other concerns during pregnancy, including pelvic/hip bony abnormalities and lower extremity contractures that often interfered with vaginal delivery and necessitated C-sections. A recent descriptive case series[45] reported on 17 women with spina bifida who experienced 29 pregnancies. Of these pregnancies, 23 (79%) progressed to live offspring. Although none of the offspring had spina bifida, three (13%) were born with other significant congenital malformations, such as tetralogy of fallot. About one-half of the women required C-sections, with postpartum complications that required prolonged hospitalization. Of the vaginal deliveries, 37% had postpartum complications. Almost one-half of the infants also had postnatal complications that included hypoglycemia, jaundice, respiratory distress syndrome, and tachypnea. Back pain and sciatica were common complaints, but in this series, no problems were encountered with ventriculoperitoneal shunts or with neurological deterioration.

Women with spina bifida are often born with renal and genitourinary abnormalities[46,47] that require urinary diversions and conscientious annual observation of renal function. During pregnancy, urinary and gastrointestinal incontinence may become problematic[48]. It is known that urinary tract infections, during any woman's pregnancy, contribute to low birth weight and preterm infants[49]. The increased incidence of urinary tract infections in women with spina bifida can put them at risk for these complications. Another high-risk condition is the possibility of conduit stomal displacement and obstruction[50]. It is also possible that obstruction and renal failure[38,51] may develop, especially if the abdomen is further compromised by exaggerated spine curvature. C-section rates are high and are estimated to be about 42%[48] to 50%[45]. Performance of a C-section in a woman with one or more prepregnancy urinary and/or bowel diversion procedure(s) demands collaboration between obstetrical and urological management.

### EFFECTS OF PREGNANCY ON SPINA BIFIDA SEQUELAE

As stated, women with spina bifida have recognized secondary conditions that may influence pregnancy. Concomitantly, the physiologic process of pregnancy and the effects of the growing fetus may exacerbate spina bifida sequelae. Kyphoscoliosis may become more pronounced, ventriculoperitoneal/

ventriculoatrial shunts may malfunction and require revision, and genitourinary diversions may fail. The frequency of seizures may increase in women who have a history of seizures. Many anticonvulsant medications are contraindicated in pregnancy and their discontinuation further exacerbates this problem.

Liakos et al.[48] retrospectively reported on 70 women with hydrocephalus and prepregnancy shunt placement, seven of whom required shunt revisions during pregnancy. Other shunt-related problems included increased seizures, headaches, and abdominal pain. Complications of shunt failures were associated with ventriculoperitoneal shunts. A total of 138 pregnancies resulted in 105 live births (77%). The spontaneous abortion rate was relatively high (20%) and about 11% of the infants had congenital abnormalities, although none with spina bifida.

Shunt failures have occurred in many women[52,53,54,55,56]. On the other hand, studies have described a high undiagnosed prevalence of increased intracranial pressure[57], arrested hydrocephalus (AH) syndrome, and Chiari/hydrosyringomyelia complex[58] in adults (male and female) with spina bifida who have never been shunted. Obviously, these unrecognized conditions could have serious implications in pregnancy management or delivery procedures.

The prevalence of spine alignment abnormalities has been reported to be 43 to 50%[32,46] of patients with spina bifida. In addition, lower extremity contractures and pelvic deformities can affect 70% of these individuals. Skeletal abnormalities[51] predispose to maternal respiratory compromise, cardiac failure[52], alterations of center of gravity, and difficulties with labor and delivery progression. These problems may necessitate a C-section or forceps delivery[53]. In addition, epidural anesthesia may be more difficult to perform or may be less effective due to the inability to gain access and satisfactorily envelop appropriate sensory neurons displaced in the spinal canal.

Other complications may occur during pregnancy. Decubiti may develop as the woman becomes less mobile, with inability to change her positioning. Respiratory compromise from the growing fetus may result in pneumonias during pregnancy. Significant back pain and possible fractures of the pelvic bones may occur from the pressures exerted on those areas. Functional limitations are frequent as the woman is unable to transfer or continue to ambulate. In the case of the latter, postpartum ambulation status may be affected.

### QUESTIONS OF GENETIC AND ENVIRONMENTAL RISKS TO OFFSPRING

Many women with spina bifida who become pregnant express concerns for fetal genetic risks. Questions of genetic and environmental risks to the offspring of women with spina bifida are significant considerations. Retrospective case studies have focused attention on the occurrence of spina bifida and other congenital abnormalities in the infants of women with spina bifida. The majority of genetic investigation, however, has examined the role of the inheritance and/or environmental factors that contribute to the risk of able-bodied women having a child with spina bifida.

Previous studies[59,60] suggest a recurrence risk for neural tube deficits/spina bifida of 4 to 7% in offspring of women with spina bifida. Historical data provide evidence that although the risk is high for the offspring of women with spina bifida to develop congenital or developmental abnormalities, the actual cases of offspring with spina bifida have not been documented. Reasons are multifactorial and possibly related to the higher miscarriage and spontaneous abortion rate, improved genetic counseling, and intrapartum amniocentesis evaluation.

Blatter et al.[61] examined medical histories and risk factor questionnaires of 210 children with spina bifida and 671 "reference" able-bodied children. The study corroborated previous investigations[62,63,64] that a positive family history of spina bifida increases the risk of spina bifida in siblings. Further studies have attempted to elucidate maternal genetic markers for spina bifida. Investigations have focused on a variety of possible mutations involved in fetal folate metabolism, such as reduced folate carrier gene[65] and methionyl tetrahydrofolate reductase gene[66]. Although not proven, it is reasonable to conclude that if the contribution of genetic variance contributing to spina bifida is "carried forward" in the woman with spina bifida, the likelihood of her transferring it to her offspring increases[67]. It has been proven that nutritional deficits of folic acid during pregnancy increase the risk of having a child with spina bifida[68,69]. The implications of appropriate prenatal supplementation of folic acid for women with spina bifida are indisputable. However, the exact mechanisms of the interactions among these other environmental contributions in combination with genetic alterations are unknown.

### MENOPAUSE

No studies have been published that explore the effects of menopause transition in women with spina bifida. One reason for this deficit is that in the past, the life span of these women was shorter and menopause was seldom reached. Premature menopause and all the ramifications of hormonal changes need to be studied in this patient population, including hormonal levels and possible supplementation based on the woman's individual needs.

With menopause, the woman's body will respond to the lack of estrogen. Decreasing estrogen alters bone metabolism (osteoporosis)[70], skin integrity[71]**,** urological maintenance, and cardiovascular health[72]. For women with spina bifida, menopause may promote acceleration of further decline of the systems. For example, osteoporosis from immobility and lack of neurologic development exists in individuals with spina bifida. A state of bone loss can predispose to worsening scoliosis and vertebral and lower extremity fractures. A balanced bladder or healthy skin may be altered as estrogen decreases and alters mucosal or dermal integrity.

### HEALTH MAINTENANCE

Some studies[73,74,75] have examined the experiences of women with physical disabilities (of many types) that have reported reproductive health maintenance and screening problems. The general consensus is that women with disabilities are less likely to receive pelvic exams, Pap smears, mammograms, and sexual health information. Not only is the impact of this problem on women with spina bifida yet to be studied, the current screening recommendations lack evidence of adaptation to this patient population.

### CONCLUSION

In general, little research has examined gynecologic and reproductive concerns of women with spina bifida and, in many cases, women with similar disabilities. Although studies of women with other neurologic disabilities can help to guide us in the care of women with spina bifida, little evidence-based research exists for this particular population. Future controlled prospective research studies are needed and should examine issues related to puberty, sexual, and gynecologic issues throughout the life span and pregnancy.
